# Editorial: Exploring small molecule probes: advancements and applications in pharmacological research

**DOI:** 10.3389/fphar.2025.1652445

**Published:** 2025-07-09

**Authors:** I-Ju Yeh

**Affiliations:** Department of Anesthesia, Indiana University School of Medicine, IN, United States

**Keywords:** small molecule probes, drug discovery, targeted therapy, high-throughput screening, proteome profiling

Small molecule probes are powerful tools that have transformed pharmacological research by enabling precise interrogation of biological systems. Their ability to selectively modulate protein function, track cellular pathways, and uncover new therapeutic targets continues to expand the frontiers of both basic and translational science. Recognizing the importance and evolving nature of this field, we launched the Research Topic *Exploring Small Molecule Probes: Advancements and Applications in Pharmacological Research* to provide a platform for emerging innovations and applications of chemical probes across diverse biological contexts.

This Research Topic brought together researchers with expertise in chemical biology, structural pharmacology, and drug discovery to explore how small molecule probes can be utilized to better understand the proteome, identify disease-relevant targets, and support therapeutic development. The accepted studies in the research topics collectively illustrate the creativity, technical advancement, and translational potential enclosed in small molecule probe research today.

One notable contribution, “*Utilizing small molecules to probe and harness the proteome by pooled protein tagging with ligandable domains”*, introduces an elegant strategy to systematically study protein-small molecule interactions. By integrating pooled protein tagging with ligandable domains, this approach offers a scalable way to profile the ligandability of the proteome, which could significantly accelerate probe development pipelines and functional annotation of understudied proteins.

In another study, researchers reported the *development of a high-throughput acoustic droplet ejection mass spectrometry assay and a solid-supported membrane (SSM)-based electrophysiological assay to study the pharmacological inhibition of SLC1-A3, -A2 and -A1*. These assays provide complementary, label-free platforms to investigate the pharmacology of excitatory amino acid transporters, which play crucial roles in neurotransmission and are increasingly being explored as therapeutic targets in neuropsychiatric and neurodegenerative disorders.

Targeting oncogenic drivers remains a central goal of small molecule drug discovery, and this Research Topic includes two compelling examples. The article *Discovery of Novel and Highly Potent Small Molecule Inhibitors Targeting FLT3-ITD for the Treatment of Acute Myeloid Leukemia Using Structure-Based Virtual Screening and Biological Evaluation* describes the identification of new FLT3-ITD inhibitors through virtual screening and biological validation. This work not only underscores the utility of *in silico* techniques in lead discovery but also offers promising scaffolds for further preclinical development.

Similarly, the study titled *Discovery of novel PARP1/NRP1 dual-targeting inhibitors with strong antitumor potency* introduces a multitargeted approach to cancer therapy. By designing dual inhibitors that engage both PARP1 and NRP1, the authors present a strategy that could improve efficacy and overcome resistance mechanisms associated with monotherapy. Such dual-targeting molecules exemplify the expanding capabilities of small molecule probes to modulate complex signaling networks in cancer.

The final article, *Radiosynthesis and in-vitro identification of a molecular probe 131I-FAPI targeting cancer-associated fibroblasts*, highlights the intersection of chemical probe development and diagnostic imaging. Fibroblast activation protein (FAP) has emerged as a relevant target in tumor stroma, and the synthesis of a radiolabeled FAPI probe enables selective detection of cancer-associated fibroblasts. This study offers valuable tools for imaging applications and further supports the idea that molecular probes can bridge therapeutic and diagnostic objectives.

Together, these contributions emphasize the versatility and impact of small molecule probes across disciplines—from proteomics and neuropharmacology to oncology and nuclear medicine. Importantly, they demonstrate how methodological advances (e.g., new assay systems, virtual screening platforms, and radiochemical synthesis) are driving the next-generation of pharmacological tools.

Looking forward, continued progress in this field will require integration of multidisciplinary approaches, including computational chemistry, structural biology, systems pharmacology, and medicinal chemistry. Further attention to probe selectivity, cellular permeability, and *in vivo* stability will enhance the translational value of these tools. As small molecule probes continue to evolve from research reagents to potential therapeutics, the importance of open, collaborative platforms such as this Research Topic cannot be overstated.

We would like to thank all authors who contributed to this Research Topic, the reviewers who provided thoughtful and constructive feedback, and the editorial team for supporting this initiative. We hope this Research Topic serves as a valuable resource for the community and inspires further innovation in the design and application of small molecule probes in pharmacological research.

